# Clinicopathologic features of ovarian neoplasms with emphasis on borderline ovarian tumors: an institutional perspective

**DOI:** 10.1186/s13104-016-2015-5

**Published:** 2016-04-06

**Authors:** Atif Ali Hashmi, Zubaida Fida Hussain, Aneel Roy Bhagwani, Muhammad Muzzammil Edhi, Naveen Faridi, Syed Danish Hussain, Mehmood Khan

**Affiliations:** Department of Histopathology, Liaquat National Hospital and Medical College, Karachi, Pakistan; Liaquat National Hospital and Medical College, Karachi, Pakistan; Intern, Liaquat National Hospital and Medical College, Karachi, Pakistan; Intern, Dhaka Medical College, Dhaka, Bangladesh

**Keywords:** Ovarian tumors, Pakistan, Mucinous ovarian tumors

## Abstract

**Background:**

Ovarian cancer is the most lethal gynecologic malignancy and it represents third most common malignancy in Karachi (after breast and oral cancer). Due to lack of well established cancer registry in our country, changing trends of ovarian tumors has not been determined. Therefore we aimed to establish the current trends and classification of ovarian tumors in our setup according to latest WHO guidelines.

**Methods:**

We retrospectively analyzed 162 cases of ovarian tumors that underwent surgical resection from January 2009 till December 2014. Specimens were received in histopathology department, Liaquat National hospital and cases were examined by senior histopathologists and classified according to latest WHO guidelines. Various histopathologic parameters including capsular invasion, omental and lymph node meatstasis along with uterine and fallopian tube involvement were determined apart from tumor type and grade.

**Results:**

Mean age at diagnosis was 35.8 years (± 15.5). surface epithelial tumors were most common, 109 cases (67.2 %) followed by germ cell tumors, 44 cases (27.1 %) and sex cord stromal tumors, 8 cases (4.9 %). Serous tumors were most common surface epithelial tumors with 90 % benign morphology. On the other hand, mucinous tumors showed a higher percentage of borderline and malignant features (16.7 and 14.6 % respectively). Higher incidence of capsular invasion and omental metastasis was noted in endometroid and serous carcinoma compared to mucinous tumors.

**Conclusions:**

We noted a higher frequency of young age ovarian cancers in our set up. Serous and endometroid carcinomas were found to be associated with adverse prognostic factors like capsular invasion and omental metastasis. Moreover a significantly higher proportion of ovarian tumors constitute mucinous histology including borderline tumors. Whether this represents a changing trend towards biology of these tumors in this part of the world needs to be uncovered by further studies.

## Background

Ovarian cancer is the most lethal gynecologic malignancy and owing to lack of early symptoms and screening protocol, it is detected late in the clinical course of the disease. The worldwide incidence rate is 6.3/100,000 women and is the seventh most common cancer diagnosis. Ovarian cancer is the third most malignancy in Karachi (after breast and oral cancer) with an age standardized incidence rate of 10.9 % [1]. The high incidence of ovarian cancer in this part of the world may be due to uncovered BRCA mutations in our population, but this is yet to be fully determined.

One of the unique characteristics of ovarian tumors are their capacity to undergo peritoneal metastasis in the absence of invasive growth in the ovary [13]. This has led to the concept of borderline tumors with a strict histologic criteria not influenced by its metastatic counterpart in the peritoneum. Due to lack of well established cancer registry in our country, changing trends of ovarian tumors has not been determined. Therefore we aimed to establish the current trends and classification of ovarian tumors in our setup according to latest WHO guidelines.

## Methods

We retrospectively analyzed 162 cases of ovarian tumors that underwent surgical resection from January 2009 till December 2014. During this period of time, all cases with a provisional diagnosis of ovarian tumors requiring surgical treatment were included in the study. Out of the 162 cases included in the study, 35 had a provisional or frozen section diagnosis of malignancy and thus underwent bilateral oophorectomy along with total abdominal hysterectomy and omental, pelvic lymph nodes sampling. On the other hand, 127 cases were benign and thus underwent oophorectomy only. All specimens were received in histopathology department, Liaquat National hospital. After gross examination, representative sections were stained with hematoxylin and eosin stains. Immunohistochemical stains were applied when necessary. Cases were examined by senior histopathologists and classified according latest WHO guidelines. Various histopathologic parameters including capsular invasion, omental and lymph node metastasis along with uterine and fallopian tube involvement were determined apart from tumor type and grade.

## Results

Mean age at diagnosis was 35.8 years (± 15.5). Surface epithelial tumors were most common, 109 cases (67.2 %) followed by germ cell tumors, 44 cases (27.1 %) and sex cord stromal tumors, 8 cases (4.9 %). Serous tumors were most common surface epithelial tumors with 90 % benign morphology. On the other hand mucinous tumors showed a higher percentage of borderline and malignant features (16.7 and 14.6 % respectively). Malignant surface epithelial tumors were significantly seen to be more common in older age group compared to their benign and borderline counterparts (Table 1). Malignant surface epithelial tumors also tend to had larger sizes compared to benign and borderline cases (Table 2). Table 3 shows various histopathologic parameters of ovarian tumors. Higher incidence of capsular invasion and omental metastasis was noted in endometroid and serous carcinoma compared to mucinous tumors. On the other hand, high frequency of ovarian capsular involvement was seen in malignant germ cell tumors and fibrosarcoma. Uterine and cervical involvement was also noted in a subset of serous and mucinous carcinomas.

**Table 1:** Age distribution of ovarian tumors

	Age groups			
	<20 years	20–40 years	40–60 years	>60 years
	n (%)	n (%)	n (%)	n (%)
Surface epithelial tumor				
Benign	14 (17.1)	44 (53.7)	18 (22)	6 (7.3)
Borderline	2 (25.0)	4 (50)	2 (25)	0 (0)
Malignant	0 (0)	7 (38.9)	4 (22.2)	7 (38.9)
Germ cell tumor				
Benign	3 (8.1)	27 (73.0)	5 (13.5)	2 (5.4)
Malignant	4 (66.7)	2 (33.3)	0 (0)	0 (0)
Sex cord stromal tumor				
Benign	1 (14.3)	3 (42.9)	0 (0)	3 (42.9)
Malignant	0 (0)	1 (100.0)	0 (0)	0 (0)
Metastatic tumor				
Malignant	0 (0)	0 (0)	1 (100)	0 (0)

**Table 2:** Tumor size distribution of ovarian neoplasms

	Tumor size	Tumor size category			
	Mean	<5 cm	5–10 cm	10–20 cm	>20 cm
		n (%)	n (%)	n (%)	n (%)
Surface epithelial tumor					
Benign	10.31	15 (18.3)	36 (43.9)	27 (32.9)	4 (4.9)
Borderline	19.56	0 (0)	0 (0)	6 (66.7)	3 (33.3)
Malignant	19.08	1 (5.6)	3 (16.7)	6 (33.3)	8 (44.4)
Germ cell tumor					
Benign	10.41	4 (10.5)	19 (50)	13 (34.2)	2 (5.3)
Malignant	14.15	1 (16.7)	0 (0)	4 (66.7)	1 (16.7)
Sex cord stromal tumor					
Benign	15.07	0 (0)	2 (28.6)	4 (57.1)	1 (14.3)
Malignant	23.0	0 (0)	0 (0)	0 (0)	1 (100.0)
Secondary malignancy					
Malignant	6.0	0 (0)	1 (100)	0 (0)	0 (0)

**Table 3:** Histopathologic characteristics of borderline and malignant ovarian tumors

	Capsular invasion	Omental metastasis	Pelvic lymph node metastasis	Uterine involvement	Cervical involvement	Contalateral ovarian involvement	Fallopian tube involvement
	n (%)	n (%)	n (%)	n (%)	n (%)	n (%)	n (%)
Borderline							
Serous tumours	0 (0)	0 (0)	0 (0)	0 (0)	0 (0)	0 (0)	0 (0)
Mucinous tumours	0 (0)	0 (0)	0 (0)	0 (0)	0 (0)	0 (0)	0 (0)
Malignant							
Serous tumours	2 (50)	1 (25)	1 (25)	1 (25)	0 (0)	0 (0)	1 (25)
Mucinous tumours	1 (14.3)	2 (28.6)	1 (14.3)	1 (14.3)	1 (14.3)	0 (0)	1 (14.3)
Endometrioid carcinoma	3 (75)	2 (40)	0 (0)	1 (20)	0 (0)	0 (0)	3 (60)
Transitional cell car	0 (0)	1 (100)	0 (0)	0 (0)	0 (0)	0 (0)	0 (0)
Dysgerminoma	1 (50)	1 (50)	0 (0)	1 (50)	1 (50)	0 (0)	0 (0)
Yolk sac tumor	1 (100)	0 (0)	0 (0)	0 (0)	0 (0)	0 (0)	0 (0)
Fibrosarcoma	1 (100)	0 (0)	0 (0)	0 (0)	0 (0)	0 (0)	0 (0)

## Discussion

Ovarian cancers are the most common malignancies of the female genital tract and the 6th leading cancer in USA. It is estimated that the lifetime risk of developing an ovarian cancer among females is 1 in 70 [1]. Ovarian carcinomas are a heterogeneous group of neoplasms showing diverse morphological characteristics, mechanisms of pathogenesis and clinical features [2]. According to morphological features, ovarian tumors are classified as surface epithelial tumors, germ cell tumors, sex cord stromal tumors and metastatic tumors. The mean age of patients with these tumors is 63 years with 80 % of the tumors occurring after 45 years of age [3]. However our study shows that the mean age of tumors has significantly lower and more cases of ovarian tumors were seen in females at comparatively younger ages. Approximately 40 % of malignant surface epithelial ovarian tumors were found in an age group of 20–40 years. Due to lack of cancer registries in our country, the demographics of ovarian cancer are poorly understood. Various studies conducted in our population revealed a lower age group distribution of breast cancer in this part of the world due to probable BRCA mutations may play a role in young age ovarian cancer as well [4]. Liaquat National Hospital is one of the largest tertiary care centre of the province and represents both rural and urban patient population of all age groups, therefore lower age distribution of ovarian cancer in our study don’t represent hospital demographics.

The distribution of these tumors shows variations in the incidence globally. Surface epithelial tumors were found to be the most common ovarian tumors. The incidence of these tumors in our study (67.2 %) is comparable to a previous study done in the same region [5]. However studies done from other areas of Pakistan have shown slight variation in the incidence of these tumors. The frequency of surface epithelial tumors from Punjab province was found to be 57 % [6] and KPK province was found to be 76.5 % respectively [7]. Surface epithelial tumors were followed by germ cell tumors and sex cord stromal tumors. The frequency of these tumors was found to be 27 and 4.9 % respectively in our study comparable to the literature from other parts of the country [6, 7].

In our study majority of these tumors were benign and only a few cases of borderline and malignant category were found. The most common tumors were benign serous tumors accounting for 90 % of the serous surface epithelial tumors and 44.9 % of all surface epithelial tumors, mostly presenting in females of the reproductive age group. However our study is almost consistent with the findings of study done by Ghartimagar et al. which showed an incidence of benign serous surface epithelial tumors 49.4 % among of all surface epithelial tumors [8].

Benign serous tumors were followed by mucinous and endometroid tumors. It was noted that although majority of mucinous tumors were benign there was a higher incidence of mucinous tumors of the borderline category. Literature shows that the majority of the mucinous tumors are benign, 20 % borderline and 5 % invasive [9]. A recent review by J. Prat showed similar results indicating that majority of mucinous tumors i.e., 80 % are benign remainder are borderline and 3–4 % of the tumors being malignant [10]. On the other hand, our study showed similar results for the benign and borderline categories but there was a higher incidence of invasive mucinous neoplasms, 14.6 % compared to 5 % cited in literature. This shows that the incidence of invasive mucinous tumors is much higher in our population as compared to literature from the west.

Germ cell tumors comprised of 27 % of all the ovarian neoplasms in our study. Of which majority of these tumors (86 %) were mature teratomas whereas rests of the tumors were malignant germ cell neoplasms. This proportion of germ cell tumors corresponded to the results of a study done in India by Mondal et al. however the similar study showed a slightly higher numbers of other germ cell tumors which were not present in our study [11].

Sex cord stromal tumors accounted for 4.9 % of all ovarian neoplasms and majority of tumors comprised of benign tumors. The incidence of these tumors corresponds with literature as being the least common subtype of ovarian tumors [12, 13]. The main limitation of this study is that it is a single center experience and thus referral bias may be a limiting factor.

## Conclusions

We found a higher frequency of young age ovarian cancers in our setup. Serous and endometroid carcinomas were found to be associated with adverse prognostic factors like capsular invasion and omental metastasis. Moreover a significantly higher proportion of ovarian tumors constitute mucinous histology including borderline tumors. Whether this represents a changing trend towards biology of these tumors in this part of the world needs to be uncovered by further studies.
